# Seroprevalence of hantavirus and Borrelia burgdorferi in Düzce (Turkey) forest villages and the relationship with sociodemographic features

**DOI:** 10.3906/sag-1807-160

**Published:** 2019-04-18

**Authors:** Nida AKAR, Emel ÇALIŞKAN, Cihadiye Elif ÖZTÜRK, Handan ANKARALI, Özge KILINÇEL, Şükrü ÖKSÜZ, İdris ŞAHİN

**Affiliations:** 1 Department of Medical Microbiology, Faculty of Medicine, Hacettepe University, Ankara Turkey; 2 Department of Medical Microbiology, Faculty of Medicine, Düzce University, Düzce Turkey; 3 Department of Biostatistics and Medical Science, Faculty of Medicine, İstanbul Medeniyet University, İstanbul Turkey; 4 Medical Microbiology Laboratory, Düzce Atatürk State Hospital, Düzce Turkey

**Keywords:** *Borrelia burgdorferi*, Düzce, forest villages, hantavirus, seroprevalence

## Abstract

**Background/aim:**

Hantavirus and *Borrelia burgdorferi* are two zoonotic agents that pose a great risk especially for people living in forest areas. This study aimed to investigate the seroprevalence of hantavirus and *B. burgdorferi *in forest villages of Düzce and its relationship with sociodemographic features.

**Materials and methods:**

The presence of immunoglobulin M (IgM) and immunoglobulin G (IgG) antibodies against hantavirus and *B. burgdorferi* in serum samples was investigated via enzyme-linked immunosorbent assay (ELISA). Hantavirus IgG and *B. burgdorferi* IgM and IgG positivity was then validated by western blot (WB) method.

**Results:**

During the analyses,****193 serum samples were tested. Eleven (6%) cases of hantavirus IgM was found positive by ELISA. Six (3%) cases of hantavirus IgG, 3 (2%) cases of *B. burgdorferi* IgM, and 12 (6%) cases of *B. burgdorferi* IgG were found positive by WB. *Borrelia burgdorferi* IgG positivity was found to be higher in the 46–70 age group and it was seen that the positivity was the highest among those who lived in mud-brick houses.

**Conclusion:**

This study was the first to determine the frequency of hantavirus in the study region and it includes current data for *B. burgdorferi.* Consequently, it is recommended that similar studies be carried out on rodents in all the regions at risk.

## 1. Introduction

Hantaviruses, members of the family* Bunyaviridae*, are enveloped viruses that have a single-stranded negative-polarity RNA genome consisting of three segments: L (large), M (medium), and S (small) (1,2). They cause two types of syndromes in humans: hemorrhagic fever with renal syndrome (HFRS) and hantavirus pulmonary syndrome (HPS). Moreover, a number of hantavirus types cause different disease patterns in various regions with different clinical courses (3). The main reservoirs of the hantavirus are various rodents and insectivores. Infection in humans results from ingestion of urine, feces, and other extracts of infected animals through the skin and mucous membranes or by inhalation of aerosols in infected air (4). In Turkey, the first cases were reported in the provinces of Zonguldak, Bartın, Giresun, and Ordu and a few cases were reported from other provinces (5,6).

The gold standard method for laboratory diagnosis of hantavirus infections is the focus reduction neutralization test (FRNT). However, this method is expensive and time- and labor-consuming, and it requires biosafety level 3 laboratory conditions. For these reasons, the FRNT can only be applied in certain centers. Therefore, the diagnostic approach is usually based on serological tests such as enzyme-linked immunosorbent assay (ELISA), immunoblot, or immunofluorescence. The immunoblot test is more sensitive and specific than ELISA and it is used to confirm the ELISA results (7). 

*Borrelia burgdorferi*, a member of the family* Spirochaetacea*, is a highly mobile microorganism with spindle and vibratory movements. It has a diameter of 0.2–0.5 µm, a length of 8–30 µm, and between 3 and 30 soft coils (8,9). The spirochete passes on to people through the bite of the genus *Ixodes* of hard ticks and causes Lyme disease (LD) (10). The rate of *B. burgdorferi* in seroprevalence studies carried out in different regions of Turkey varies between 6% and 35.9% (11). 

The Centers for Disease Control and Prevention recommended a two-stage procedure for serological diagnosis of LD. First, a sensitive test, usually an ELISA, should be performed, and if the results are positive or borderline, it should be validated by the immunoblot test, which has high specificity (12).

Forest areas are the habitats of rodents and insectivores that play a role in the transmission of hantaviruses. These areas are also the habitats of ticks that contribute to the spread of *B. burgdorferi*, thus putting the inhabitants of these areas at risk for hantavirus and *B. burgdorferi* infections.

This study aimed to gather information on the seroprevalence of hantavirus and *B. burgdorferi *in forest villages of Düzce, to evaluate the level of public knowledge about them, and also to raise awareness of the protective measures necessary for those living in regions at risk of diseases caused by this virus and bacterium.

## 2. Materials and methods

This study was carried out with the approval of the Düzce University Ethics Committee for Clinical Investigations (decision dated 27.06.2016 and numbered 2016/16).

### 2.1. Population and sampling of the study

The study population included inhabitants of forest villages in Düzce, an area that provides a suitable living environment for rodents and insects effective in the transmission of hantavirus and* B. burgdorferi *to humans. According to the data from the Provincial Directorate of Population and Citizenship Affairs, the population of forest villages is recorded as 5001.

The size of the population, as determined by the power test, was taken as 80%, with the possibility of type I error as 5%. The average frequency in the literature was also taken into consideration. According to this evaluation, the appropriate number of blood samples should be at least 150. 

### 2.2. Sample collection

The study included individuals living in forest villages of Düzce who were aged between 18 and 70 and who agreed to give blood samples during the field study between the dates of 28.06.2016 and 10.07.2016. All the participants in the study gave their informed consent. Venous blood samples were obtained from these participants and a survey was conducted for each participant who gave blood samples. The samples were brought to the Medical Microbiology Laboratories of Düzce University Application and Research Hospital in compliance with cold chain procedures. After 10 min of centrifugation at 3000 rpm, the separated serum samples were stored at –80 °C until the time of processing.

### 2.3. Method

In this study, ELISA (Euroimmun, Lübeck, Germany) was used to detect antibodies for the type of hantavirus and *B. burgdorferi *IgM and IgG in the serum samples. 

Anti-Hanta Profile 1 (Euroimmun) strips impregnated with recombinant nucleocapsid antigens of Puumala (PUUV), Dobrava (DOBV), and Hantaan (HTNV) were used to validate the positive results of the serum samples examined via the Anti-Hantavirus IgG ELISA. The strips were evaluated using the EUROLineScan program (Euroimmun) according to the manufacturer’s recommendations. The results were found to be positive and negative. 

The serum samples that were found positive by the anti-*Borrelia* IgM ELISA were validated by the Anti-*Borrelia* IgM WB test (Euroimmun) and the serum samples that were found positive by the Anti-*Borrelia* IgG ELISA were validated using the Anti-*Borrelia* IgG WB test (Euroimmun). Western blot test kits included strips that were impregnated with electrophoretically separated *B. burgdorferi* antigens. The strips were evaluated with the EUROLineScan program (Euroimmun) according to the manufacturer’s recommendations. The results were both positive and negative. 

### 2.4. Statistical analyses

The descriptive values of the data obtained were given in terms of mean, standard deviation, number, and percentage frequencies. The relationships between the results of ELISA and the WB validation test along with other features were examined using the Fisher–Freeman–Halton test. In addition, the diagnostic successes were calculated. The statistical significance level was set as 5%. SPSS 18 (SPSS Inc., Chicago, IL, USA) was used for the calculations. 

## 3. Results

The serum samples of 193 individuals collected from 11 villages in Düzce Province were included in the study. Of the participants, 103 (53%) were female and 90 (47%) were male, and the mean age was calculated as 47.4 ± 13.5 years.

The results of the ELISA screening and the WB validation results of the positive screenings are shown in Table 1.

**Table 1:** The results of the ELISA screening and the WB validation results of the positive screenings [n (%)].

	Hantavirus		B. burgdorferi	
Method	IgM *	IgG	IgM	IgG
ELISA-positive	11 (6)	13 (7)	27 (14)	21 (11)
WB-positive	-	6 (3)	3 (2)	12 (6)

* Hantavirus IgM ELISA-positive patients were not studied by the WB method.

Of the 6 cases found to be hantavirus IgG-positive with the WB test, 5 (83%) were found to be PUUV-positive and 1 (7%) to be DOBV-positive.

When the screening results of hantavirus IgM positivity via ELISA were evaluated, no statistical difference was determined between the groups. In the 18–45 age group, it was found that the positivity was higher compared to the 46–70 age group and it accounted for 63% of all hantavirus IgM positivity. The positivity rate of forestry workers was higher than those who do not work in the forest. When the hantavirus IgG results via WB were examined, although no statistical difference was determined, it was seen that the positivity rates were higher in the 46–70 age group. The positivity rates were also higher for those with an education level below middle school and with a monthly income of 1500 TL and below. Similarly, forestry workers had higher positivity rates than the others. The results of the evaluation of hantavirus-positive samples with the methods of ELISA and WB are shown in Table 2.

**Table 2:** The evaluation results of hantavirus positivity in samples using ELISA and WB.

Hantavirus							
	IgM ELISAn (%)	P	IgG ELISA n (%)	P	IgG WB n (%)	P	Totaln
Sex							
Male	6 (7)	0.588	6 (7)	0.971	5 (6)	0.099	90
Female	5 (5)		7 (7)		1 (1)		103
Age distribution							
18–45	7 (8)	0.212	4 (5)	0.356	1 (1)	0.239	83
46–70	4 (4)		9 (8)		5 (5)		110
Education status							
Below middle school	6 (4)	0.342	10 (7)	0.796	5 (4)	1.000	136
Middle school	4 (9)		3 (7)		1 (2)		43
University	1 (7)		0 (0)		0 (0)		14
Monthly income level (TL)							
<1500	8(6)	1.000	10 (7)	1.000	5 (3)	1.000	143
≥1500	3 (6)		3 (6)		1 (2)		50
Job groups							
Farmer	6 (4)	0.064	8 (6)	0.591	3 (2)	0.306	136
Laborer	1 (3)		3 (9)		2 (6)		32
Civil servant	4 (16)		2 (8)		1 (4)		25
House structure							
Reinforced concrete	8 (7)	0.854	9 (7)	0.573	5 (4)	0.078	122
Wooden	3 (5)		3 (5)		0 (0)		62
Mud-brick	0 (0)		1 (11)		1 (11)		9
Working in forestry							
Yes	7 (6)	0.918	8 (7)	0.989	5 (4)	0.412	120
No	4 (6)		5 (7)		1 (1)		73
Total	11 (6)		13 (7)		6 (3)		193

In the evaluation made by the WB method, it was detected that 3 patients who were *B. burgdorferi* IgM-positive were in the 46–70 age group and they were engaged in livestock farming and forestry. Only 1 of these 3 patients was *B. burgdorferi* IgG-positive. In addition, the positivity rate was higher in patients with tick bite history (8%) than those without history (3%). Furthermore, in evaluations with the WB method, it was observed that 10 (83%) of 12 patients who were *B. burgdorferi* IgG-positive were in the 46–70 age group, their education level was below middle school, their monthly income was less than 1500 TL, and they had tick bite histories. When the type of materials used in house construction was considered, those living in mud-brick houses had the highest positivity levels. The results of *B. burgdorferi* positivity with ELISA and WB methods are shown in Table 3.

**Table 3:** The evaluation results of samples with B. burgdorferi positivity via ELISA and WB.

B. burgdorferi									
	IgM ELISA n (%)	P	IgM WB n (%)	P	IgG ELISA n (%)	P	IgG WBn (%)	P	Totaln (%)
Sex									
Female	13 (13)	0.678	1 (1)	0.599	14 (14)	0.249	9 (9)	0.144	103
Male	14 (16)		2 (2)		7 (8)		3 (3)		90
Age distribution									
18–45	15(18)	0.156	0 (0)	0.261	4 (5)	0.019	2 (2)	0.057	83
46–70	12 (11)		3 (3)		17 (15)		10 (9)		110
Education status									
Below middle school	16 (12)	0.302	3 (2)	1.000	18 (13)	0.158	10 (7)	0.684	136
Middle school	9 (21)		0 (0)		2 (5)		2 (5)		43
University	2 (14)		0 (0)		0 (0)		0 (0)		14
Monthly income level (TL)									
<1500	19 (12)	0.634	3 (1)	0.570	19 (11)	0.069	10 (7)	0.734	143
≥1500	8 (16)		0 (0)		2 (4)		2 (4)		50
Job groups									
Farmer	15 (11)	0.156	2 (1)	0.652	17 (13)	0.713	11 (8)	0.220	136
Laborer	7 (22)		1 (3)		2 (6)		0 (0)		32
Civil servant	5 (20)		0 (0)		2 (8)		1 (4)		25
House structure									
Reinforced concrete	18 (15)	0.718	1 (1)	0.363	14 (12)	0.045	8 (7)	0.049	122
Wooden	9(15)		2 (3)		4 (7)		2 (3)		62
Mud-brick	0 (0)		0 (0)		3 (33)		2 (22)		9
Tick bite history									
Yes	14 (12)	0.403	2 (2)	0.799	16 (14)	0.156	9 (9)	0.128	67
No	13 (17)		1 (1)		5 (6)		3 (3)		126
Working in forestry									
Yes	20 (17)	0.203	3 (3)	0.291	10 (8)	0.159	6 (5)	0.375	120
No	7 (10)		0 (0)		11 (15)		6 (8)		73
									
Livestock farming									
Yes	21 (13)	0.239	3 (2)	0.330	20 (12)	0.322	11 (7)	0.503	165
No	6 (21)		0 (0)		1 (4)		1 (4)		28
Total	27 (14)		3 (2)		21 (11)		12 (6)		

## 4. Discussion

Recently, studies on hantavirus epidemiology and data on the distribution of the virus in Turkey have been increasing. *Apodemus flavicollis*, *Apodemus agrarius*,* Rattus norvegicus*,**and *Rattus rattus,* which are reservoirs of the Dobrava, Hantaan, and Seoul viruses, and *Clethrionomys glareolus*, which is a reservoir of the Puumala virus, are reported to be common in Turkey. These findings suggest the possibility that there could be endemic areas in the country infected with these viruses (13). 

When the antibody responses in hantavirus infections are examined, the IgM antibody appears relatively early after the infection. Titers of IgM begin to decrease a few weeks after the infection and can be detected in blood for 6–8 months. IgG antibodies appear shortly after IgM or simultaneously with IgM. Titers of IgG in blood may remain elevated for many years (14). In the present study, in addition to the detection of seroprevalence, the positivity levels of both hantavirus IgG and IgM were investigated by the ELISA method in order to determine cases in the acute phase. In this study, hantavirus IgM positivity was found to be 6%, which is similar to the findings of Montgomery et al. (15) in South America. However, the hantavirus species with the HPS factor are endemic to South America, while the hantavirus species with the HFRS factor are frequently observed in Turkey. The participants were informed that hantavirus infection may have symptoms such as fatigue, fever, muscle pain, headache, dizziness, abdominal pain, diarrhea, vomiting, nausea, and shortness of breath and they were advised that those who displayed these symptoms should contact the study team. There was no contact with the team in this period, which suggested that the patients were asymptomatic.

Because each rodent subfamily carries a phylogenetically different species of hantavirus, variations occur in the seroprevalence of endemic hantavirus species in each country and the infections caused by these species (16). Oldal et al. (17) found that the prevalence of hantavirus IgG in forestry workers in Hungary was detected as 4.6% with the WB test. Engler et al. (18) investigated the prevalence of hantavirus IgG in military personnel and blood donors in Switzerland and found the rate to be 0.3% in blood donors and 0.3% in the whole group via the WB method. In Turkey, Gozalan et al. (19) found the seroprevalence of hantavirus IgG in Giresun Province to be 3.2% by using the WB method. In this study, the seroprevalence of hantavirus IgG was determined to be 3% with the WB method. When these seroprevalence rates were compared with other studies, the ratios of forestry workers and those living in forest villages were higher than in other studies. It is known that hantavirus could be transmitted to humans through inhalation when it is spread via aerosol formation from rodent feces and urine (3). This suggests that living in forested areas or working as a forestry worker increases the risk of hantavirus infection depending on the hantavirus-carrying rates of rodents and insectivores in the region.

The IgG antibody positivity of DOBV and PUUV types, though with a higher frequency of PUUV, was detected in a study carried out in Bartın, in which the WB test was used to identify the cases of HFRS (20). In another study performed in Bartın, the DOBV hantavirus in rodent species was studied by Oktem et al. (21) and the presence of the DOBV type in tissues of *Apodemus flavicollis* and *Apodemus uralensis* was demonstrated via reverse transcription polymerase chain reaction. Similarly, the present study detected IgG positivity for hantavirus PUUV and DOBV. This similarity could have resulted from the comparable vegetation and climatic conditions and the geographical proximity of Düzce and Bartın. It is thought that studies on the seroprevalence of hantavirus should be carried out for the rodent population in Düzce Province as well.

Socioeconomic level and poor living conditions could be important factors in the transmission of infectious diseases. Gozalan et al. (19) demonstrated that living in mud-brick or wooden houses is a significant risk factor for the transmission of hantaviruses. The seroprevalence of hantavirus IgG was found to be higher in those living in mud-brick houses, those with a monthly income less than 1500 TL, and those with a low education level in this study. These findings are thought to suggest that the socioeconomic level is a risk factor for hantavirus infection, because it affects hygiene habits and living conditions.

LD is the most common tick-borne disease in some parts of North America, Asia, and Europe. *Ixodes* ticks live in Turkey, which suggests that LD might be widespread in the country (22). The first LD cases were reported in Turkey in 1990, and as the numbers of cases increased, epidemiological studies began to be carried out. In the studies conducted in Turkey, the Lyme seropositivity rate in agriculture-related risk groups was found to be 6%–35.9% (23–25). 

In LD, the humoral response begins with the formation of IgM and can usually be detected in the serum in 2 to 4 weeks, peaking at 8 to 10 weeks, and then it decreases gradually. IgG antibodies can be detected in the serum in 6 weeks after the onset of the infection, peaking at 4 to 6 months, and it remains detectable in the serum for years (26). Some patients could be seronegative during the acute infection. Despite adequate treatment of LD and clinical improvement, IgM can rarely remain positive for years (27). In the present study, IgM levels of *B. burgdorferi* were also investigated in order to determine early cases besides seroprevalence. As *B. burgdorferi* IgM-positive patients did not develop any symptoms, it is thought that they had the disease in asymptomatic form. In addition, only 1 of the 3 IgM-positive patients was also IgG-positive and there were no clinical signs. This could be due to the rare persistence of IgM despite clinical improvement.

Generally, LD does not discriminate between age, sex, or race. However, individuals who live or work in areas where ticks are widespread are at high risk (28). When the seroprevalence rates were examined for North American, European, and Asian countries where LD is prevalent, the prevalence of *B. burgdorferi* IgG varied between 4% and 13% (29–31). In Turkey, the positivity of *B. burgdorferi* IgG was found to be 0.9% in the provinces of Van and Manisa, 4.6% in Bolu, and 6% in Düzce (by the present study) (32–34). The difference between the regions could be attributed to geographical location, climatic conditions, and species of ticks living in the region. The high level of *B. burgdorferi *seropositivity in the northern part of Turkey compared to the other regions suggests that this region is endemic for *B. burgdorferi*.**It is observed that the seroprevalence of *B. burgdorferi* IgG in Düzce has increased by 5% since 2007, when it was evaluated with the WB method (25). The vegetation and climatic conditions of the western Black Sea region are considered to be significant factors for the increase in the numbers of ticks, which are vectors of *B. burgdorferi*, and for their spreading the disease*.* In addition, the level of positivity has increased since the study was carried out in 2007, which indicates that the level of public knowledge in the region is insufficient to protect the population. Studies show that LD is a disease that affects children, adults, and the elderly. *B. burgdorferi* IgG positivity is known to last a lifetime, so its high presence in those of advanced age in this region suggests that living in the region for a long time increases the risk of infection.

Studies on the seroprevalence of *B. burgdorferi* have reported that the seroprevalence of LD is low in groups with high socioeconomic and educational levels (28,35). Bucak et al. (33) found that the prevalence was higher in the illiterate population than in the literate population. In the current study, it was observed that *B. burgdorferi* IgG positivity decreased as the level of education increased. Those with a high level of education are more conscious of ticks and the diseases transmitted by them, and those with a high socioeconomic level have more access to personal hygiene and care facilities. For these reasons, the prevalence was thought to be low in these groups. This is supported by the high level of positivity detected among those living in mud-brick houses as compared to those living in wooden and reinforced concrete houses.

In this study and in other studies (25,32,33,35), it was observed that the positivity of* B. burgdorferi *was higher among people who had a history of tick bite; however, the occurrence of positivity in those without a history of tick-bite is a reminder that there are means of transmission other than tick bites. In addition, as the positivity of *B. burgdorferi* IgG persists throughout the lifetime, forgotten childhood contacts with ticks could be another important factor. 

In the present study, there were some samples that were detected positive for the antibodies of hantavirus IgG and *B. burgdorferi* IgM and IgG by ELISA but negative by western blot. The reason might be the cross-reacting of genetically similar infectious agents in the ELISA method. Moreover, the ELISA method used to diagnose LD could be false positive due to the HIV infection, infectious mononucleosis, and cross-reactions occurring in lupus or rheumatoid arthritis (36).

In conclusion, this study revealed that hantavirus and *B. burgdorferi* antibodies are found in populations with a low income and a low education level, which shows that awareness-raising activities should be carried out to prevent infections caused by these agents. Moreover, it is recommended that studies on hantavirus seroprevalence should be carried out on the rodent population in the region as hantavirus PUUV and DOBV positivity was detected in the area.

## Acknowledgments 

We would like to thank Professor Mehmet Ali Öktem from the Dokuz Eylül University Faculty of Medicine, Department of Medical Virology under the Department of Medical Microbiology, for his invaluable help for the validation and evaluation of the results. This study was supported by Düzce University Scientific Research Projects numbered 2016.04.01.424 and was prepared from the first author’s thesis study, of which the second author was the thesis advisor.
